# Role of *Legionella pneumophila* outer membrane vesicles in host-pathogen interaction

**DOI:** 10.3389/fmicb.2023.1270123

**Published:** 2023-09-25

**Authors:** Ayesha Ayesha, Franklin Wang-Ngai Chow, Polly Hang-Mei Leung

**Affiliations:** Department of Health Technology and Informatics, The Hong Kong Polytechnic University, Kowloon, Hong Kong SAR, China

**Keywords:** outer membrane vesicles, Legionnaires’ disease, *Legionella pneumophila*, host-pathogen interaction, LCV, CAP, HAP

## Abstract

*Legionella pneumophila* is an opportunistic intracellular pathogen that inhabits artificial water systems and can be transmitted to human hosts by contaminated aerosols. Upon inhalation, it colonizes and grows inside the alveolar macrophages and causes Legionnaires’ disease. To effectively control and manage Legionnaires’ disease, a deep understanding of the host-pathogen interaction is crucial. Bacterial extracellular vesicles, particularly outer membrane vesicles (OMVs) have emerged as mediators of intercellular communication between bacteria and host cells. These OMVs carry a diverse cargo, including proteins, toxins, virulence factors, and nucleic acids. OMVs play a pivotal role in disease pathogenesis by helping bacteria in colonization, delivering virulence factors into host cells, and modulating host immune responses. This review highlights the role of OMVs in the context of host-pathogen interaction shedding light on the pathogenesis of *L. pneumophila*. Understanding the functions of OMVs and their cargo provides valuable insights into potential therapeutic targets and interventions for combating Legionnaires’ disease.

## 1. Introduction

Lower respiratory infections are the fourth leading cause of death worldwide (WHO, 2020). Pneumonia is the most common type of lower respiratory tract infection clinically represented by pulmonary alveoli inflammation and caused by a diverse class of pathogens including viruses, bacteria, parasites, and fungi. Among many kinds of pneumonia infections, Legionnaires’ is a severe manifestation which is caused by the bacteria “*L. pneumophila*.” Recent studies have identified *Legionella* spp. among the four most common microbial causes of hospitalizations due to community-acquired pneumonia (CAP) (Stout and Yu, 1997; Woodhead, 2002). Although it is primarily associated with CAP, Legionnaires’ disease is also observed in healthcare settings as hospital-acquired pneumonia (HAP) when water systems are poorly managed in hospitals.

*Legionella pneumophila*, the causative agent of Legionnaires’ disease was discovered in 1977 after an outbreak of pneumonia in Philadelphia. Since then it has been linked to many outbreaks caused by improper management of artificial water management. As per the WHO report of September 2022, the overall death rate of Legionnaires’ disease is 5–10% among all infected individuals and 40–80% in immunocompromised patients. Proper case management can reduce the mortality rate in immunocompromised to 5–30% (WHO, 2022). Perhaps as a result of a variety of variables such as host risk factors, late diagnosis and poorly maintained artificial water systems incidences of Legionnaires’ disease are increasing every year. To control incidences and improve disease management, understanding the host-pathogen interaction is vital, potentially paving the way for the development of targeted therapeutics and interventions.

Recent research has highlighted bacterial extracellular vesicles as mediators of communication among bacteria and, between bacteria and host cells. These vesicles contribute to the development of disease and regulate the host immune response. Extracellular vesicles are lipid bilayer structures released by living organisms of all domains. The name of these vesicles varies depending on the type of organism and the nature of originating cells. As gram-negative bacteria like *L. pneumophila* contain an outer membrane, vesicles shed by gram-negative bacteria are named outer membrane vesicles (OMVs). Although OMVs were first discovered and visualized by Narayan in 1966, the mechanisms of OMVs regulation and functions are still unclear (Chatterjee and Das, 1967). This review summarizes the pathogenesis of *L. pneumophila*, role of OMVs in host-pathogen interactions, and addresses gaps in the study of *L. pneumophila* OMVs highlighting their importance in the host-pathogen interaction.

## 2. Legionella pneumophila

Intracellular pathogen *L. pneumophila* is an aerobic obligate gram-negative rod that widely inhabits the freshwater environment. This organism is an opportunistic pathogen causing either mild-flu like illness known as Pontiac fever or acute form of pneumonia known as Legionnaires’ disease (Hamilton et al., 2018). Typically, the organism is found in freshwater in free form or association with biofilms. The ability of *L. pneumophila* to reproduce within biofilms offers additional defense against environmental stresses like biocides, nutrient depletion, and adverse temperatures. The organism can also infiltrate and persist intracellularly in a variety of protozoans including *Acanthamoeba, Vermamoeba*, and *Naegleria* etc. in both soil and aquatic environments (Newsome et al., 1985; Atlas, 1999; Siddiqui et al., 2021). Since the life cycle of the *L. pneumophila* generally requires endoparasitization and reproduction within eukaryotic protists like amoebae, *L. pneumophila* have also developed the ability to infect human cells, particularly the macrophages.

The typical route of transmission of *L. pneumophila* is through inhalation of contaminated aerosols. Common sources of the spread of *L. pneumophila* in communities include humidifiers, whirlpool spas, air conditioning cooling towers and, hot and cold water systems (Mondino et al., 2020). While in hospitals, infection can occur through the exposure of newborns to infectious aerosols during water deliveries and the aspiration of contaminated water by susceptible hospitalized patients (Muder et al., 1986; Franzin et al., 2001). Direct human-to-human transmission has not yet been documented (Lorry and Rubin, 2008).

Soon after being taken up by lung macrophages, *L. pneumophila* bypasses the airway defense system by evading endocytic maturation pathway, preventing phagosome-lysosome fusion and developing a niche for replication called LCV (*Legionella* containing vacuole). This LCV compartment differs from phago-lysosome compartment since it does not acidify and is formed by recruitment of vesicles from rough endoplasmic reticulum (ER) (Robinson and Roy, 2006). Following the establishment of replication niche, *L. pneumophila* secrete effector proteins to modulate host cell signaling, host membrane trafficking, ubiquitin and autophagy pathways to favor its replication inside host. *L. pneumophila* multiplying inside the LCV is knowns as the replicative phase of the infection cycle. The intracellular replication cycle within the lung cell is completed after depletion of nutrients after which *L. pneumophila* shifts toward transmissive phase by destroying the host cell and release from it. The released bacteria then spread to nearby host cells and starts a new infection cycle (Mondino et al., 2020).

One of the outstanding features of *L. pneumophila* is its ability to reproduce within different hosts. In order to successfully establish infection cycle in multiple hosts series of distinct events are required, many of which are performed by action of one or more of over 300 effector proteins of *L. pneumophila* (Lockwood et al., 2022). Like other bacteria, *L. pneumophila* uses multiple strategies to deliver proteins extracellularly and intracellularly. Particularly, secretion systems are known to facilitate the transportation of proteins and other molecules which play a vital role in their survival, virulence, and interactions with the host. *L. pneumophila* possesses secretion systems I, II, and IV which translocate effector proteins, enzymes, and virulence factors from the bacteria across the bacterial membranes, delivering them into the host cell cytoplasm. Although the T1SS is dispensable for the intracellular life cycle, but it is required for the host cell invasion mechanisms (Fuche et al., 2015). The T2SS and T4SS have received significant attention in *L. pneumophila* research because of their crucial roles in infection (Cianciotto, 2005). During *L. pneumophila* development inside the cell, the T2SS system transports more than 25 effector proteins, which are important for bacterial replication in various hosts (Cianciotto, 2009; Cianciotto, 2013). Additionally, the T2SS may play a role in biofilm formation and the dispersal of *L. pneumophila* from biofilms, which can contribute to bacterial transmission and persistence in water systems (Cianciotto, 2009; Cianciotto, 2013).

The Dot/Icm type IV secretion system significantly contributes to *L. pneumophila* virulence. Both LCV biosynthesis and intracellular replication in human and protozoans hosts require the Dot/Icm T4SS (Andrews et al., 1998; Segal and Shuman, 1999; Nagai and Roy, 2003). The *L. pneumophila* T4SS is situated at the poles of bacteria and polar secretion of effectors is necessary to alter the host endocytic pathway hence promoting bacterial survival in the host (Jeong et al., 2017). Importantly, T4SS secrete more than 330 effector proteins which regulates all intracellular life stages of *L. pneumophila* and target the fundamental cellular functions shared by protozoa and mammals (Kitao et al., 2020). Effectors Sidk, VipD, and PieE participate in *L. pneumophila* uptake and evasion from the endocytic maturation pathway. Many other effector proteins, including SidM, SidD, RaIF, and LseA, facilitate the interaction of the endoplasmic reticulum (ER) and the formation of LCV. Once the LCV is established, the organism secretes many other effectors into the host through the Dot/Icm system to hijack host cell functions including mRNA processing, the ubiquitin pathway, cell signaling, and cell death pathways (Kitao et al., 2020).

Overall, the secretion systems in *L. pneumophila* are involved in many functions like protein synthesis in host cells, the secretion of effectors to create an infectious niche, the transfer of DNA, and the secretion of autotransporters which are related to virulence and pathogenesis of the bacteria (Green and Mecsas, 2016). However, soluble secretion systems are effective over short distances as they require close physical contact between bacteria and host cells.

Apart from using secretion systems for protein delivery over short distances, *L. pneumophila*, like other gram-negative bacteria, uses outer membrane vesicles (OMVs) as long-distance delivery vehicles for transporting bioactive chemicals from bacteria to environment or host cells.

## 3. Outer membrane vesicles

### 3.1. Production

Secretion of extracellular vesicles (EVs) is a conserved mechanism found in all life forms, including bacteria, archaea, fungi, and complex eukaryotes (Deatherage and Cookson, 2012). These small lipid-membrane bounded particles, which range in size from 20–400 nm, are released from cells but lack the ability of self-replication (Maas et al., 2017; Liu et al., 2022). Bacterial vesicles production was first observed in 1960; since then, OMVs have been extensively studied in bacteria including *Escherichia coli*, *Neisseria*, *Vibrio*, *Bacteroides*, *Pseudomonas aeruginosa*, *Campylobacter jejuni* and *Actinobacillus* (Chatterjee and Das, 1967; Devoe and Gilchrist, 1973; Hoekstra et al., 1976; Logan and Trust, 1982; Nowotny et al., 1982; Grenier and Mayrand, 1987; Kadurugamuwa and Beveridge, 1995) (Toyofuku et al., 2019). In gram-positive bacteria, EVs bud from cytoplasmic membrane containing cytoplasmic contents, also known as membrane vesicles (MV) whereas in gram-negative bacteria, EV bleb from the outer membrane contain both periplasmic and cytoplasmic components referred as outer membrane vesicles (OMVs) (Brown et al., 2015; Toyofuku et al., 2019). According to recent research, gram-negative bacteria also release double- and triple-membrane vesicles in addition to OMVs which are hypothesized to be the results of bacterial cell lysis with and without bacteriophages (Toyofuku et al., 2019).

Although OMVs are produced by various bacterial species, the rates of OMV production vary among different bacterial species (Devoe and Gilchrist, 1973; Gankema et al., 1980; Wensink and Witholt, 1981; Ribeiro de Freitas et al., 2022). Moreover, the growth and nutrient conditions also influence the production and composition of OMVs within the same bacterial specie. Several studies have shown that bacteria increase vesicle production after exposure to certain antibiotics (Orench-Rivera and Kuehn, 2016). This increased OMV production can either release more enzymes such as beta-lactamases to destroy antibiotics or capture surrounding antibiotics by acting as decoys to protect bacteria against certain antibiotics. Bacterial vesiculation can also be influenced by the presence of a host. Studies reported differences in OMVs production upon exposure to host components and tissue. Using enterotoxigenic *E. coli* infection mouse model, electron microscopy observations revealed that vesicles were more abundant on ETEC cells recovered post-infection from the mouse small intestine (Ellis and Kuehn, 2010).

### 3.2. Elements/components of OMVs

Elements of OMVs purified by ultracentrifugation, filtration, or chromatography included components predominantly present in membranes, such as proteins and lipids of periplasm, as well as other cytoplasmic components (Roier et al., 2016). Protein compositions of OMVs identified the presence of outer membrane (OM) proteins, periplasmic proteins, and flagellin (OMVs derived from motile-bacteria). In lipid compositions, lipopolysaccharide (LPS) and lipoproteins were identified. Notably, a significant abundance of LPS component was observed in all OMVs originating from gram-negative bacteria (Toyofuku et al., 2019). Flagellin, LPS, OM proteins, and lipoproteins are also proposed as key players in OMV biogenesis mechanisms (Avila-Calderón et al., 2021). Oligosaccharides, also known as glycans are present in the outer membrane and can be found associated with OMVs. Since OMVs are involved in bacteria-bacteria and bacteria-host interactions, the successful docking of OMVs with other bacterial and host cell surfaces is associated with the presence of adhesive oligosaccharides within the OMVs (Knoke et al., 2020). Furthermore, the composition of oligosaccharides in OMVs varies among different bacterial strains or species. Thus, oligosaccharide present in OMVs composition can serve as a molecular fingerprint of the specific type of OMVs. However, further research and analysis are necessary to fully understand the diversity and functional significance of oligosaccharides within OMVs of different bacterial strains.

**Figure 1:**
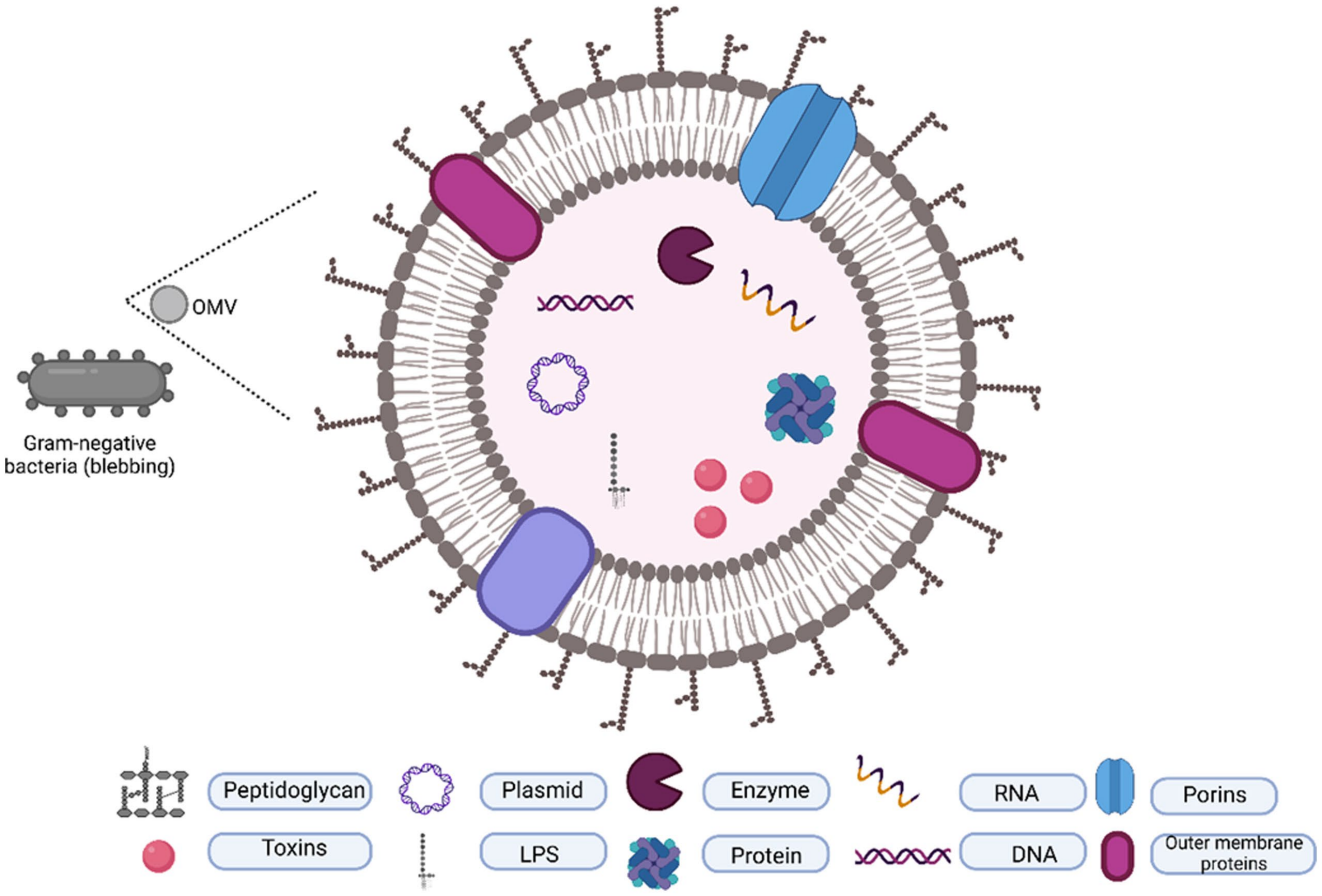
Typical composition of OMVs. OMVs are originated by outer membrane blubbing and contain a lipid layer packaged with proteins, toxins, DNA, and RNA.

Apart from flagellin, oligosaccharide, outer membrane, and periplasmic proteins, OMVs contain a variety of cargos, including nucleic acids such as plasmids, DNA, RNA, and cytosolic proteins including virulence factors (Figure 1). Although various components have been identified from the OMVs of gram-negative bacterial species, several key components remain constant in OMVs, providing us with the advantage of understanding them from the perspective of a shared origin.

## 4. Outer membrane vesicles in host-pathogen interaction

Previous studies described OMVs production as a by-product of cell lysis but recent studies demonstrate that the OMVs are actively produced by all gram-negative bacteria. These OMVs are enriched in cytoplasmic, periplasmic, virulence proteins, and specific lipids suggesting that bacteria purposefully release OMVs. There could be two reasons for this: first as a means of interaction with other bacteria and host, and secondly, for survival in stressful environments (McBroom et al., 2006).

**Figure 2:**
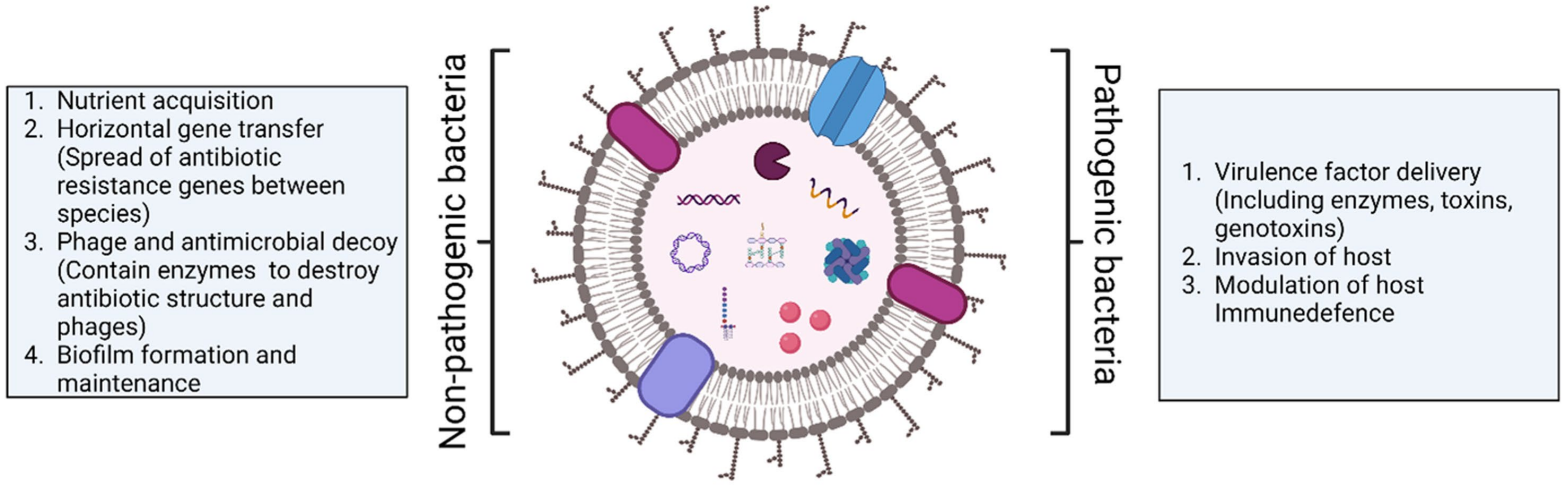
Diverse functions of OMVs released by non-pathogenic and pathogenic bacteria.

OMVs play diverse roles in both pathogenic and non-pathogenic bacteria (Figure 2). In non-pathogenic bacteria, OMVs can act as vehicles for intercellular communication among bacterial populations. OMVs carry signaling molecules, quorum sensing factors, or small RNA molecules that facilitate coordination and cooperation among bacteria. This communication helps regulate microbial community dynamics and can contribute to host homeostasis. Further, OMVs can mediate nutrient exchange among bacterial populations by carrying enzymes or nutrient-binding molecules that scavenge and acquire nutrients from the environment. This way OMVs can benefit both the producing bacteria and neighboring microbes. OMVs can also contribute to the formation and maintenance of bacterial biofilms by carrying the extracellular polymeric substances (EPS), including polysaccharides and proteins, which provide structural support to the biofilm. Recently, the beneficial role of OMVs produced by gut microbiota or commensal bacteria has also been highlighted. A study reported that OMVs released from *Akkermansia muciniphila* which colonizes the intestinal mucous layer can restore the gut microbiota balance by specifically stimulating the growth of beneficial bacteria while suppressing the proliferation of opportunistic pathogens. In addition, OMVs also improved immune functions of mucosa by switching IgM to IgA (Wang et al., 2023).

Among pathogenic bacteria, OMVs aid in the delivery of virulence factors/toxins, immune evasion, and modulation of host factors to facilitate bacterial growth. As the focus of this review is to summarize the role of OMVs in host-pathogen interaction with context to *L. pneumophila*, we will not discuss extracellular functions in detail.

### 4.1. Mechanisms of OMVs interaction with host cells

Although there is considerable evidence that OMVs can enter host cells and release their cargo to modulate host cell functions, the precise mechanisms underlying how OMVs interact with and are taken up by host cells are still not completely known. OMVs from pathogenic bacteria interact with a variety of host cells including immune cells (epithelial cells, macrophages, dendritic cells and neutrophils) and non-immune cells (endothelial cells, osteoblasts and synovial cells) (Schultz et al., 2007; Kaparakis et al., 2010; Maldonado et al., 2011; Kim et al., 2013; Lappann et al., 2013; Jäger et al., 2014; Kaparakis-Liaskos and Ferrero, 2015). Generally, there are five endocytic pathways for OMVs entry to host cells including macropinocytosis, clathrin mediated endocytosis, caveolin mediated endocytosis, lipid-raft mediated endocytosis and direct membrane fusion. Examples exist for each pathway in literature.

Evidence of macropinocytosis or actin mediated endocytosis was found when uptake of *P. aeruginosa* OMVs by airways epithelial cells was reduced after treatment with actin polymerization inhibitor, a crucial protein for actin-dependent macropinocytosis (Bomberger et al., 2009). Interestingly *P. aeruginosa* OMVs also required lipid-raft mediated endocytosis pathway for its entry to human lung epithelial cells (Bauman and Kuehn, 2009). OMVs of *L. monocytogenes* interacted with Caco-2 cells by acting-mediated endocytosis or macropinocytosis pathway (Karthikeyan et al., 2019). Clathrin-mediated endocytosis was shown to be OMVs entry route for *Brucella abortis* in human monocytes (Pollak et al., 2012), Enterohemorrhagic *E. coli* in human brain microvascular endothelial cells (HBMEC) and Caco-2 cells (Bielaszewska et al., 2013), *Aggregatibacter actinomycetemcomitans* in HeLa cell and human gingival fibroblasts (HGF) (Thay et al., 2014; Vanaja et al., 2016). Finally, OMVs are also able to uptake by host cells by direct membrane fusion which has been demonstrated in *P. aeruginosa*, *A. actinomycetemcomitans* and *L. pneumophila* by labeling OMV membranes with Rhodamine R-18 fluorescent dyes (Bomberger et al., 2009; Rompikuntal et al., 2012; Jäger et al., 2015).

However, studying interaction between OMVs and host cell is complex as the majority of pharmacological inhibitors of endocytic pathways have impact on many mechanisms, making it frequently difficult to identify the uptake process. Additionally, more than one mechanism for OMV uptake can be found within same bacterial species. OMVs content and size can also influence the uptake pathway. Details of OMVs entry mechanisms and factors effecting entry have been published elsewhere in 2020 (Caruana and Walper, 2020) and immunological effects of OMVs interactions with different host cells have also been described before (Kaparakis-Liaskos and Ferrero, 2015). Here, we discuss the interaction of *L. pneumophila* with host cell, and the subsequent immune responses triggered by the host.

## 5. *Legionella pneumophila* outer membrane vesicles

### 5.1. *Legionella pneumophila* OMVs production

Research on the biology of *Legionella* and Legionnaires’ disease for four decades has provided important insight on bacterial infection strategies. *L. pneumophila* OMVs added a new aspect to the pathogenesis of *L. pneumophila*. Flesher et al. discovered OMVs in *L. pneumophila* for the first time as membrane blebs in 1979 while studying the cell-envelope structure of bacterium using electron microscopy (Flesher et al., 1979). These vesicles were later isolated by ultracentrifugation of bacterial culture supernatants after filtration through 0.22um of the *L. pneumophila* and purity was analysed by negative staining electron microscopy (EM) and atomic force microscopy (AFM) in 2008. In microscope, they ranged in size from 100–200 nm (Galka et al., 2008). AFM and EM are considered standard method to visualize and validate the purity of OMVs fractions (Théry et al., 2018).

*L. pneumophila* produce OMVs throughout the life cycle including log phase and stationary phase and at different growth conditions including both extracellular and intracellular growth. Jung et al. confirmed intracellular production of OMVs by co-incubating *L. pneumophila* with amoeba *Dictysotelium discoideum* host for 24 h. Thin-section electron microscopy showed blebs from the *L. pneumophila* membrane surface within the *Legionella*-specific phagosome of infected *D. discoideum* host cells (Galka et al., 2008).

### 5.2. *Legionella pneumophila* OMVs binding to and uptake by host cells

The uptake of OMVs by other bacteria and host cells is a dynamic and complex process involving various pathways of interactions as described earlier. Once internalized, OMVs can deliver their cargo, including proteins, toxins, nucleic acids, antibiotic-resistance enzymes, and several other factors.

So far, the binding of *L. pneumophila* OMVs with human alveolar epithelial cells has been confirmed using confocal laser microscopy by labeling OMVs with green fluorescent anti-LPS antibody (conjugated with Alexa flour 488). After 8 h of OMVs incubation with A549 cells, confocal microscopy revealed acquisition of green color on epithelial cells surface. Further binding of OMVs to host cells also changed the cell morphology toward round shape suggesting OMVs can not only bind to host cells but also trigger significant morphological changes in host cells (Galka et al., 2008). Later *L. pneumophila* OMVs interaction with human macrophages were also observed using same Alexa-flour anti-LPS antibody. Strong green fluorescence signal was detected just after 3 min incubation of OMVs with differentiated human mononuclear cells (MNCs) and this signal intensity increased with time and with increasing OMVs protein concentration. This time and dose-dependent binding of *L. pneumophila* OMVs with human macrophages suggested that OMVs can fuse with host cells and deliver their cargo (Jäger et al., 2015). To study whether *L pneumophila* OMVs can be internalized to host cells by direct membrane fusion, Jäger et al. incubated OMVs with liposomes made up of eukaryotic phospholipids membranes. By using Fourier transform infrared spectroscopy and Förster resonance energy transfer (FRET) they found that OMV membrane material could be incorporated into liposomes model of eukaryotic membrane by direct membrane fusion (Jäger et al., 2015). However, involvement of other OMVs entry pathways is not studied in *L. pneumophila*.

Binding of *L. pneumophila* OMVs with host cells was also confirmed *in-vivo*, when lung tissue explants from healthy donors were incubated with *L. pneumophila* OMVs. Immunostaining revealed the localization of OMVs predominantly on alveolar macrophages after 24 and 48 h (Jäger et al., 2014).

### 5.3. *Legionella pneumophila* OMVs role in intracellular infection cycle

The key feature of *L. pneumophila* infections is to escape phago-lysosomal degradation and develop replicating niche. By coating latex beads with *L. pneumophila* OMVs Fernandez et al. reported that OMVs can inhibit the fusion of phagosomes containing *L. pneumophila* with lysosomes suggesting OMVs have ability of mediate the pathogenesis (Fernandez-Moreira et al., 2006). Another research found that *L. pneumophila* OMVs pre-treated macrophages had more *Legionella-*containing vacuoles (LCV) and produced less pro-inflammatory cytokines hence OMVs exposed macrophages are more susceptible for bacterial replication than unexposed macrophages (Jung et al., 2016).

### 5.4. Are *Legionella pneumophila* OMVs cytotoxic to host cells?

The extent of cytotoxicity associated with *L. pneumophila* OMVs remains unclear to date. Studies *in vitro* indicated that *L. pneumophila* OMVs are not cytotoxic to host cells. For example, in one study, the metabolic activity of PMA-differentiated human U937 macrophage cell line was studied using Alamar blue dye reduction. OMVs did not affect cell vitality after incubation with 100 μg mL^−1^ protein concentration of OMVs for 24 h (Jäger et al., 2015). Consistent with these results another research group observed non-significant reduction in growth of H292 alveolar epithelial cells after 72 h incubation with 50 μg mL^−1^ *L. pneumophila* OMVs (Galka et al., 2008). However, *in-vivo* examination of lung tissues explants after treatment with 100 μg mL^−1^ of *L. pneumophila* OMVs revealed that after attachment to alveolar epithelial cells OMVs caused damage in septa and epithelia over time suggesting OMVs can cause damage to host cells during intracellular infection (Jäger et al., 2014). Results were found opposite when *L. pneumophila* OMVs were incubated with amoeba host model *Acanthamoeba castellanii*. Surprisingly, growth of *A. castellanii* was increased after co-incubation with OMVs. As amoeba like *A. castellanii* feeds on bacteria using peptides and amino acids generally and OMVs contain significant protein hence OMVs may serve as a food source for *A. castellanii*.

The cytotoxity of OMVs to host cells may depend on multiple factors including type of host, dose of OMVs, time duration and growth phase (replicative and transmissive) of *L. pneumophila*. Further investigation is required to conclude the cytotoxity potential of *L. pneumophila*.

### 5.5. The content of *Legionella pneumophila* OMVs- what is known

While the exact mode of OMV-host interaction remains to be elucidated, studies on OMV cargos have highlighted they may contribute to bacterial pathogenesis and host immune modulation. This section highlights the probable contributions of OMV components in host-pathogen interaction and discuss the gaps in *L. pneumophila*.

#### 5.5.1. Proteins

**Figure 3:**
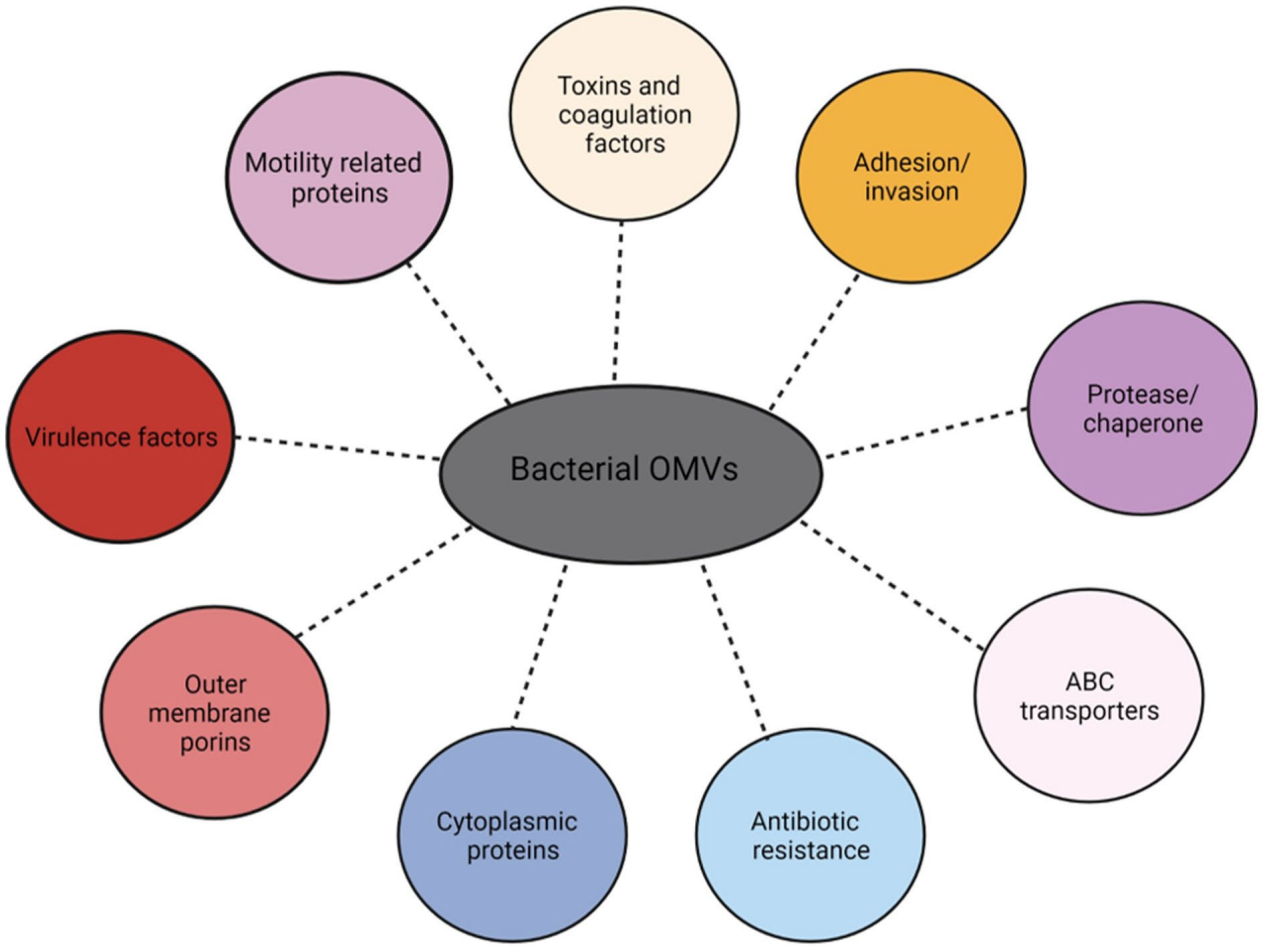
Proteins families identified in Outer Membrane Vesicles (OMVs).

Proteome analysis of gram-negative bacterial OMVs revealed that they contain diverse protein families, which include predominantly outer membrane proteins, antibiotic resistance enzymes, and other proteins (Figure 3) (Uddin et al., 2020). Following OMVs isolation, determining the protein concentration provides an estimation of the amount of OMVs present. Therefore, protein concentration of OMVs is commonly used in dosage studies of OMVs. It helps standardize the OMV dosage across experiments, ensuring consistent and reproducible results and gives direct correlation between the amount of OMVs administered and the biological response observed, such as cytokine induction, immune response, or cellular signaling.

For protein component analysis of *L. pneumophila* OMVs, OMVs were separated from bacterial supernatant fraction by ultracentrifugation (at 150,000 × *g* for 3 h) (Wai et al., 2003). Pellet obtained was suspended in Tris–HCl and termed as OMVs which was visualized by Electron microscopy in order to confirm the absence of non-OMV protein aggregates. The remaining liquid supernatant fraction was termed as SSP fraction. MALDI-TOF mass spectrometry analysis of *L. pneumophila* secretome including both pellet (OMV) and soluble supernatant fraction (SSP) revealed that OMVs contain 33 specific proteins that are not released by any other secretion systems.

**Figure 4:**
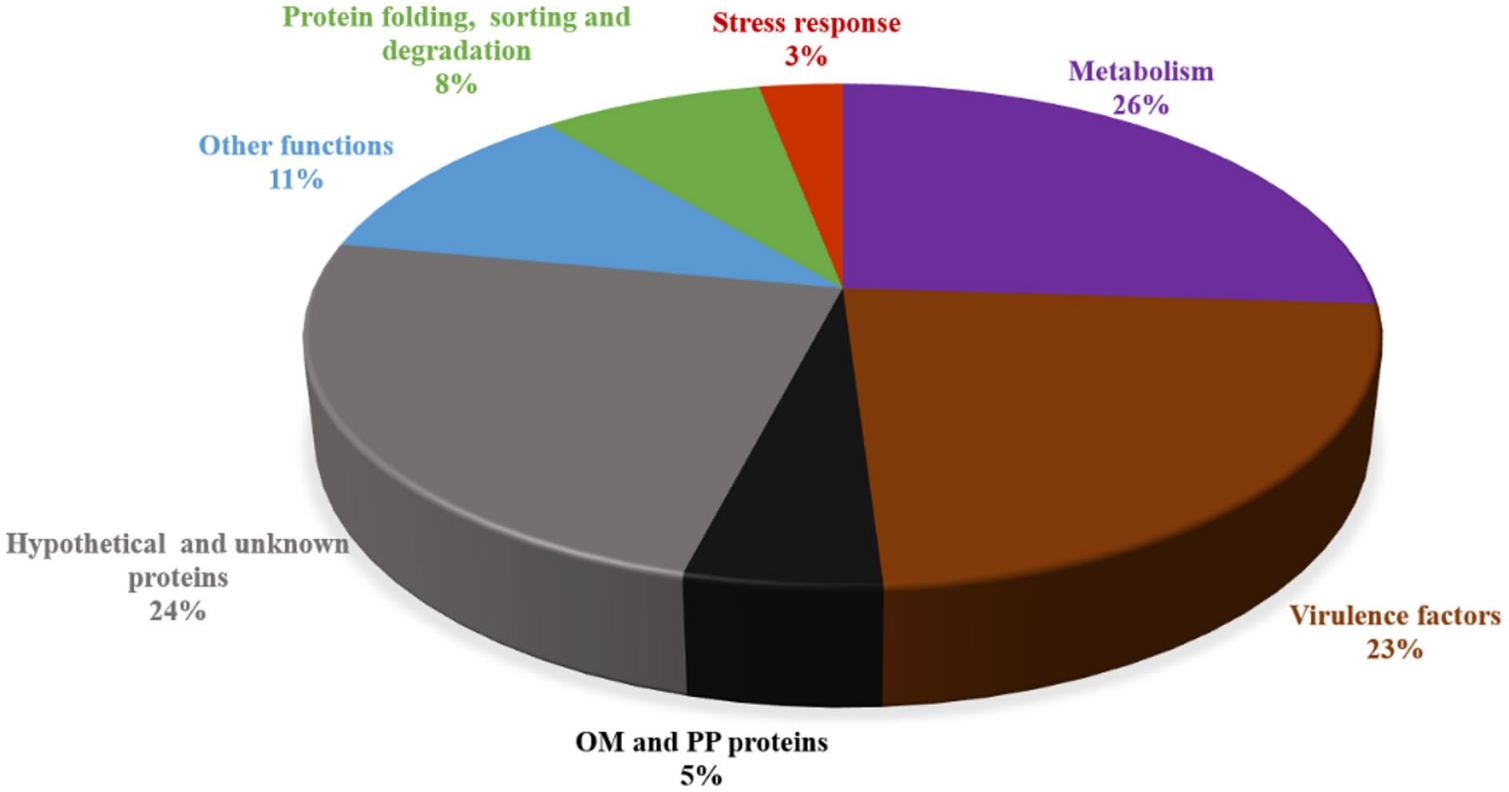
Protein families identified by KEGG pathway prediction in *L. pneumophila* OMVs.

Total 74 proteins were found in OMVs fraction of *L. pneumophila*, percentage of each functional group is described in Figure 4 (Galka et al., 2008). Eighteen out of 33 OMV-specific proteins are predicted to contribute bacterial virulence. While 41 proteins were common in both fractions and belonged to carbohydrate metabolism, energy metabolism, amino acid metabolism, and protein sorting (Galka et al., 2008). Table 1 summarizes the known functional proteins of *L. pneumophila* OMVs among the whole secretome studied by Galka et al. (2008). The presence of many virulence factors, metabolic and protein folding proteins within OMVs suggest that *L. pneumophila* may exploit OMV to deliver factors and manipulate host cell functions for its survival. Interestingly, a greater number of virulence associated proteins were found in the OMV fraction as compared to soluble supernatant fraction; out of 25 virulence factors isolated from whole secretome of *L. pneumophila*, 18 were associated with OMVs. This distribution suggests that OMVs are specific carrier of virulence-associated proteins.

**Table 1:** *Legionella pneumophila* OMVs protein families identified by 2-DE and *in silico* data analysis.

Proteins families	Gene identity (As defined in genome of *L. pneumophila* Philadelphia-1)	Predicted KEGG function in *L. pneumophila*	Description in other OMVs proteome
Protein folding, sorting or degradation			
Thiol disulphide interchange protein	lpg0123	Protein folding, sorting or degradation	*Escherichia coli* (Aguilera et al., 2014) *Neisseria meningitidis* (Vaughan et al., 2006)
L-lysine dehydrogenase (LDH)	lpg1350	Protein folding, sorting or degradation	No
DnaK chaperone protein, heat shock protein Hsp70	lpg2025	Protein folding, sorting or degradation; stress	*Francisella novicida* (Pierson et al., 2011)
Aminopeptidase	lpg2631	Protein folding, sorting or degradation	*Pseudomonas aeruginosa* (Bauman and Kuehn, 2006)
Peptidylprolyl cis-trans isomerase B (ppiB)	lpg2726	Protein folding, sorting, or degradation	*Pseudomonas aeruginosa* (Choi et al., 2011) *Acinetobacter baumannii* (Yun et al., 2018)
**Outer membrane protein and periplasmic protein**			
OmpH outer membrane protein	lpg0507	OMV biogenesis/Other functions	*Pasteurella multocida* (Fernández-Rojas et al., 2014) *Fusobacterium necrophorum* (Bista et al., 2023)
27 kDa outer membrane protein (Com1)	lpg1841	OMV biogenesis/Other functions	No
Major outer membrane protein (MOMP)	lpg2960	OMV biogenesis/Other functions	No
Rod shape determining protein (MreB)	lpg0811	Cell envelope	No
**Metabolism**			
Catalase/hydroperoxidase (KatG)	lpg0194	Energy metabolism; amino acid metabolism	*Francisella novicida* (McCaig et al., 2013)
Acetoacetate decarboxylase	lpg0672	Carbohydrate metabolism; lipid metabolism	No
Acetyl-CoA acetyltransferase (fadA)	lpg1353	Lipid metabolism; amino acid metabolism; xenobiotics biodegradation and metabolism	*Fusobacterium nucleatum* (Liu et al., 2019)
Glutamine synthetase, type I (glnA)	lpg1364	Energy metabolism; amino acid metabolism; peptidoglycan biosynthesis	No
Nucleoside diphosphate kinase (ndk)	lpg1548	Nucleotide metabolism	No
Aconitate hydratase (acnA)	lpg1690	Carbohydrate metabolism; energy metabolism	*Mycobacterium tuberculosis* (Lee et al., 2015) *Bacteroides fragilis* (Zakharzhevskaya et al., 2017)
Long-chain fatty acid transporter	lpg1810	Lipid metabolism	No
S-adenosylmethionine synthetase (metK)	lpg2022	Amino acid metabolism	No
Aspartate semialdehyde dehydrogenase (Asd)	lpg2302	Amino acid metabolism	No
Malate dehydrogenase (mdh)	lpg2352	Carbohydrate metabolism; energy metabolism	*Acinetobacter radioresistens* (Fulsundar et al., 2015) *Bacillus subtilis* (Bergsma et al., 1981) *Escherichia coli* (Aguilera et al., 2014)
GTP cyclohydrolase I (folE2)	lpg2766	Metabolism of cofactors and vitamins	*Pseudomonas syringae* (Chowdhury and Jagannadham, 2013) *Campylobacter jejuni* (Taheri et al., 2019)
Polyribonucleotide nucleotidyltransferase (Pnp)	lpg2768	Nucleotide metabolism	*Francisella novicida* (Pierson et al., 2011) *Neisseria meningitidis* (Williams et al., 2007)
Inosine 5′-monophosphate dehydrogenase	lpg2843	Nucleotide metabolism	*Staphylococcus haemolyticus* (Cavanagh et al., 2019) *Fusobacterium nucleatum* (Munshi, 2020) *Cryptococcus neoformans* (Rodrigues et al., 2008)
ATP synthase subunit A and B	lpg2984	Energy metabolism	No
Fumarylacetoacetate hydrolase	lpg2279	Amino acid metabolism; xenobiotics biodegradation and metabolism	*Moraxella catarrhalis* (Schaar et al., 2011)
Glu/Leu/Phe/Val dehydrogenase	lpg2275	Amino acid metabolism	No
Endo-1,4 beta-glucanase	lpg0482	Metabolism	No
Virulence factors			
Astacin protease (legP)	lpg2999	Virulence/pathogenesis	No
Phospholipase/lecithinase/hemolysin, lysophospholipase A	lpg2837	Virulence/pathogenesis	*Pseudomonas aeruginosa* (Kadurugamuwa and Beveridge, 1995)
IcmX (IcmY)	lpg2689	Virulence/pathogenesis	No^a^
Tail fiber protein (SclB)	lpg2644	Virulence/pathogenesis	No^a^
SdeD (LaiF)	lpg2509	Virulence/pathogenesis	No^a^
TPR repeat protein	*lpg2222*	Virulence/pathogenesis	*Porphyromonas gingivalis* (Mantri et al., 2015) *Fibrobacter succinogenes* (Arntzen et al., 2017) *Francisella tularensis* (Klimentova et al., 2019)
LaiE	lpg2154	Virulence/pathogenesis	No^a^
Phospholipase C	lpg1455	Virulence/pathogenesis	*Pseudomonas aeruginosa* (Bomberger et al., 2009) *Acinetobacter baumannii* (Jha et al., 2017)
Flagellin (FliC)	lpg1340	Virulence/pathogenesis	*Pseudomonas aeruginosa* (Bauman and Kuehn, 2006) *Campylobacter jejuni* (Jang et al., 2014)
Major acid phosphatase (Map)	lpg1119	Virulence/pathogenesis	No
Chitinase	lpg1116	Virulence/pathogenesis	*Francisella novicida* (Pierson et al., 2011) *Bacillus thetaiotaomicron* (Elhenawy et al., 2014)
ecto-ATP diphosphohydrolase II	lpg0971	Virulence/pathogenesis	No
Macrophage infectivity potentiator (Mip)	lpg0791	Virulence/pathogenesis	*Neisseria meningitidis* (Williams et al., 2007)
Hsp60, 60 K heat shock protein (HtpB)	lpg0688	Virulence/pathogenesis	*Piscirickettsia salmonis* (Oliver et al., 2016) *Campylobacter jejuni* (Lindmark et al., 2009) *Neisseria meningitidis* (Ferrari et al., 2006)
Icmk (DotH)	lpg0450	Virulence/pathogenesis	No^a^
IcmE (DotG)	lpg0451	Virulence/pathogenesis	No^a^
Phosphatidylcholine hydrolysing phospholipase	lpg0502	Virulence/pathogenesis	No
Zinc metalloprotease (ProA, Msp)	lpg0467	Virulence/pathogenesis	*Vibrio cholerae* (Rompikuntal et al., 2015)
Stress response			
Cold shock protein CspE	lpg2825	Stress	*Klebsiella pneumoniae* (Zhang et al., 2021)
DNA binding stress protein	lpg0689	Stress	*Pseudomonas syringae* (Kulkarni et al., 2014)

^a^Specific effector proteins of *L. pneumophila*.

Composition and functions of these OMV proteins are not studied yet during intracellular infection of *L. pneumophila*. However, the literature on over all bacterial infection suggests that each set of OMV proteins may play a role in the pathogenesis of bacteria, as mentioned in the following discussion.

##### 5.5.1.1. Membrane associated proteins

Nearly all OMVs are loaded with OM (outer membrane) proteins and PP (periplasmic proteins). OM proteins primarily responsible for OMVs formation may also perform additional functions like adhesion to host cells (Lee et al., 2007). OmpH found in *L. pneumophila* OMVs is a major structural protein associated with membrane phospholipids, essential for outer membrane formation and hence may play role OMV formation (Khemiri et al., 2008). Another protein found in *L. pneumophila* OMVs is com1 which was annotated in 2008 as OM protein because of its similarity to Com1 of *Coxiella burnetii* outer membrane protein, later localization studies in 2011 confirmed that com1 is periplasmic protein and was designated as DsbA2. Literature shows that DsbA2 is responsible for assembly of Dot/Icm T4SS during *L. pneumophila* infection. This 27 kDa protein is also responsible for *L. pneumophila* motility and viability (Hendrix et al., 1993; Jameson-Lee et al., 2011). MOMP (Major outer membrane protein) which is considered as important virulence factor of *L. pneumophila* was also found in OMVs. During infection of *L. pneumophila* MOMP binds with complement proteins and facilitate the bacterial uptake *via* complement receptors. It has also been shown to reduce phagocytosis of macrophage and increase the expression of IL-10, NOD2, MCP-1, and RIP2 (Bellinger-Kawahara and Horwitz, 1990; Yang et al., 2021).

##### 5.5.1.2. Invasion and adhesion factors

OMVs are also enriched with proteins and enzymes that enhance their invasive properties, thereby promoting OMV internalization at the host interface (McMillan et al., 2021). They can be found either localized in outer membrane of OMVs or in lumen of OMVs. *L. pneumophila* OMVs contain phospholipase, chitinase and astacin protease in lumen. Astacin proteases belong to the family of metalloproteinases and are involved in protein degradation. In *L. pneumophila*, astacin proteases are associated with tissue damage and host cell invasion. These proteases can degrade extracellular matrix components, such as collagen and elastin, facilitating tissue penetration and dissemination within the host. They also contribute to the activation of host cell signaling pathways, modulating the host immune response, and promoting bacterial survival (Bond and Beynon, 1995; Banerji et al., 2008). Similarly, phospholipases are enzymes that hydrolyse phospholipids, breaking them down into their constituent parts. In *L. pneumophila*, phospholipases are involved in altering the host cell membrane and promoting both invasion of bacterium in host cell and escape of the bacterium out the Legionella-containing vacuole (LCV) into the host cell cytoplasm. By disrupting the host cell membrane, phospholipases also contribute to the cytotoxic effects of *L. pneumophila* infection (Istivan and Coloe, 2006). Chitinases are enzymes that degrade chitin, a complex polysaccharide found in the protozoa and biofilms. In *L. pneumophila*, chitinase plays a role in the bacterium’s environmental invasion, survival and virulence in protozoa or biofilm matrices (Chen et al., 2020). Other protein found in *L pneumophila* OMVs, the heat shock protein Hsp60 is found crucial for bacterial adhesion and invasion in HeLa cell model (Garduño et al., 1998).

##### 5.5.1.3. ABC transporters and metabolism enzymes

OMV-associated ABC transporters and metabolic enzymes play a crucial role in bacterial survival during nutritional deficiency (Nevot et al., 2006; Lappann et al., 2013). Interestingly, *L. pneumophila* OMVs contain a great percentage of metabolic enzymes. Metabolic enzymes are crucial for energy production and the biosynthesis of essential molecules required for bacterial growth and survival. The TCA cycle, where these enzymes function, is vital for the bacterium’s adaptation to various environmental conditions and its ability to replicate within host cells during infection. Acetyl-CoA produced by FadB- FadA (acetyl-CoA acetyltransferase) mediate degradation of fatty acids and feeds directly into the TCA cycle. Asd (aspartate semialdehyde dehydrogenase) is important in the biosynthesis of amino acids and Asd mutant of *L. pneumophila* was unable to survive in amoeba and macrophage (Harb and Kwaik, 1998). Role of OMV-packaged metabolic enzymes in bacterial pathogenies is still unclear. They may influence the metabolic state of neighboring cells, potentially modulating host cell signaling pathway and host responses, scientific evidence of which is missing. Understanding the role of these enzymes in OMVs can provide valuable insights into *L. pneumophila* physiology, virulence, and their interactions with the host.

##### 5.5.1.4. Virulence factors

Many virulence factors are associated with bacterial membrane vesicles of pathogenic bacteria which either damage host cells directly or modulate host immune defence (Grenier and Mayrand, 1987; Kadurugamuwa and Beveridge, 1995; Kolling and Matthews, 1999; Horstman and Kuehn, 2000; Haurat et al., 2011; Prados-Rosales et al., 2011; Coelho et al., 2019)*. Several virulence factors were found in L. pneumophila OMVs*. A virulence protein of *L. pneumophila* KatG detoxifies antibacterial reactive oxygen compounds produced by host macrophages (Manca et al., 1999). Another major virulence protein Mip, is a stable homodimer. Mip can bind to collagen IV based on its PPIase activity and therefore enables *L. pneumophila* to transmigrate over tissue barriers of lung epithelial cells (Wagner et al., 2007). It can also promote proliferation of bacteria in LCV by inhibiting the acidification of phagosome consequently reducing the phagocytosis of macrophages (Shen et al., 2022). IcmX protein plays role in the establishment of LCV and pore formation in macrophage cell membrane (Shevchuk et al., 2011). Another virulence factor Zinc metalloprotease or Msp have been reported to cause destruction in lung tissue explants by collagen IV degradation (Scheithauer et al., 2021). Intracellular studies are needed to confirm the contributions of these virulence factors.

##### 5.5.1.5. Protein degradation and stress response

Function of cold-shock and DNA binding stress proteins are not well studied in *L. pneumophila*. But literature on other bacteria shows that cold-shock proteins can sense and respond to temperature changes and other environmental stresses and allows bacteria to thrive in various challenges (Keto-Timonen et al., 2016). Many proteins were identified in *L. pneumophila* OMVs which participate in protein folding, sorting and degradation. As these protein folding chaperons are involved in the regulation of membrane dynamics and curvature, they might get packed inside during biogenesis of OMVs. However, their exact functions in OMVs are still uncertain. OMVs can also serve to remove toxic compounds such as misfolded protein by this function group of proteins under environmental stresses (Rollauer et al., 2015; Schwechheimer and Kuehn, 2015).

#### 5.5.2. Nucleic acids associated with OMVs

Diverse genetic materials have been found in association with OMVs including; chromosomal DNA, Plasmid DNA, phage/viral DNA, mRNA, rRNA, sRNA and tRNA (Yaron et al., 2000; Biller et al., 2014; Ho et al., 2015; Blenkiron et al., 2016).

##### 5.5.2.1. RNA cargo

OMVs contain variety of RNAs including mRNA, rRNA, sRNA and tRNA (Domingues and Nielsen, 2017). Interestingly, while the function of OMVs-associated DNA in disease pathogenesis remains uncertain, OMVs-associated RNA plays a substantially significant role during disease pathogenesis. Bacterial RNA can be packaged and transferred to other bacteria and host *via* OMVs (Tsatsaronis et al., 2018; Lee, 2019). Table 2 summarizes the studies on association of RNA with OMVs and their functions including *L. pneumophila*. Among all groups of RNA, sRNA has been found more significant in host-pathogen interaction which range in size from 20 to 200 nucleotide. Although there are largely unknown about packing, delivery stability, and host selection they have shown many regulatory mechanisms by binding to protein targets and modify their functions (Lalaouna et al., 2013; Koeppen et al., 2016). This binding can have a wide range of negative effects on the cell’s metabolic, apoptotic, and immunomodulatory processes. These sRNA of bacteria are similar to miRNA and small interfering RNAs (SiRNA) of eukaryotes in function. Hence during host-pathogen interaction they mimic host miRNA, which plays key role in gene expression regulation, and modulate host cell functions including immune responses.

**Table 2:** Summary of published articles reporting RNA association with OMVs.

Organism	OMV-associated RNA studied	Study approach	Function studied	Reference

*E. coli* strain 536	Complete RNA profile (rRNA, tRNAs, small RNAs, and mRNA)	RNA-sequencing	OMV RNA uptake by the bladder epithelial cells	Blenkiron et al. (2016)
*P. aeruginosa*	Short RNA (sRNA)	RNA-sequencing	One candidate (sRNA52320) Reduced IL-8 secretion in primary human airway epithelial cells Modulated OMV-induced KC cytokine secretion and neutrophil infiltration in mouse lung	Koeppen et al. (2016)
*S. enterica* serovar *Typhimurium*	Complete RNA profile (OMVs were enriched in mRNA and non-coding RNA)	High throughput sequencing	Not assessed	Malabirade et al. (2018)
*Actinomycetemcomitans*	msRNAs (miRNA-size, small RNAs)	Northern blot and RT-qPCR	msRNA (A.A_20050) decreases IL-15, IL-13 and IL-5 secretion in Jurkat T-cells	Choi et al. (2017)
*P. gingivalis*	msRNA (miRNA-size, small RNAs)	Northern blot and RT-qPCR	msRNA (P.G_45033) decreases IL-15, IL-13 and IL-5 secretion in Jurkat T-cells	Choi et al. (2017)
*Treponema denticola*	msRNA (miRNA-size, small RNAs)	Northern blot and RT-qPCR	msRNA (T.D_2161) decreases IL-15, IL-13 and IL-5 secretion in Jurkat T-cells	Choi et al. (2017)
*Vibrio cholerae*	Small non-coding RNA gene- vrrA	Mutation analysis	*vrrA* mutant Overproduces OmpA porin leading to increased OMV production increased ability of bacteria to colonize the intestines of infant mice	Song et al. (2008)
*L. pneumophila*	The top 20 enriched sRNA in OMVs were identified (2 further studied) RsmY is analog to host miRNA144 (it regulates RIG-I expression) 2. tRNA-Phe targets the UTR of the *irak1*	RNA-sequencing	RsmY reduced the RIG-I and IFN-*β* response expression in the host. tRNA-Phe downregulated IRAK1 expression	Sahr et al. (2022)

So far, two studies have been done on *L. pneumophila* OMV-associated small RNAs. One comprehensive study identified OMV-associated very small RNAs (vsRNAs <16 nt) in 5 different bacterial species including *L. pneumophila*. RNA-seq of vsRNA revealed their abundance within OMVs along with thermodynamically stable tRFs (transfer RNA fragment). Presence of tRFs and their bioinformatic analysis by BLASTN, RNA hybrid, DIANA-microT suggest that they are eukaryotic miRNA analogues and target human mRNAs. Gene function analysis on tRFs targets by PANTHER described that they have diverse targets like cell differentiation, B cell chemotaxis, metal ion binding, and, MAP kinase activity and, regulation of cellular response to stress and macrophage colony-stimulating factor production etc. (Diallo et al., 2022). This phenomenon suggests that small RNAs packaged in *L. pneumophila* OMVs can influence the transcriptome profile of neighboring host cells during infection. Another RNA-seq study revealed that *L. pneumophila* translocate small RNAs (sRNA) by OMVs which are eukaryotic analogous. These sRNAs target host defence signaling pathways by binding to the UTR of RIG-I, IRAK1 and cReI and finally downregulating the IFNβ production (Sahr et al., 2022). Thus, a noteworthy aspect of communication between *L. pneumophila* pathogen and host is direct miRNA-like regulation of the expression of the innate immune response. Surprisingly, the discovered “bacterial miRNAs” serve two purposes: as trans-kingdom signaling molecules as well as being crucial for bacterial own survival. For example, regulatory RNA called RsmY is believed to control the life cycle *L. pneumophila*, and tRNA-Phe to be involved in protein production. It is important to find out how the sRNAs of *L. pneumophila* and other bacteria contained in OMVs affect eukaryotic cells as well as whether they share any common strategies for modulating the host immune response. Further research on the delivery of sRNAs through OMVs and their influence on host-pathogen interactions is anticipated. Due to their immunomodulatory abilities, OMVs may be the ideal vehicle for the delivery of sRNAs that target certain host genes. These numerous variables and processes are still difficult to understand and are leading to a new understanding of host-pathogen interactions.

### 5.6. The content of *Legionella pneumophila* OMVs- what needs to be known

#### 5.6.1. Lipids

The lipid composition of OMVs is found consistent across gram-negative bacterial species except some geometric changes (Silhavy et al., 2010; Gnopo et al., 2020). They serve two functions. First, they participate in the biogenesis of OMVs. Deformation of the bacterial outer membrane is necessary for OMV production and this deformation is controlled by regulating the concentration and structure of individual phospholipid and lipid A molecules (McMahon and Gallop, 2005). Second, lipids of OMVs play a role in immune response regulation. Lipid A, an endotoxin that contributes to the amphipathic base structure of LPS, is a microbe-associated molecular pattern (MAMP) which is recognized by eukaryotic pattern recognition receptors (PRRs). In response to contacts with gram-negative bacteria, PRRs regulate inflammatory reactions including host immunity, and cell death (Loppnow et al., 1989; Gioannini et al., 2004; Simpson and Trent, 2019). In addition to lipid A, cardiolipins are essential outer membrane components. These tetra-acylated di-phosphatidylglycerols also engage TL4/MD-2 receptor to activate or modulate host immune response (Mileykovskaya and Dowhan, 2009; Chandler and Ernst, 2017).

The lipidome profile of *L. pneumophila* OMVs has not been characterized yet. However, cell envelope of *L. pneumophila* is described in detail. Like other gram-negative bacteria, *L. pneumophila* also contain outer membrane made up of inner phospholipids leaflet and outer lipopolysaccharides (LPS) leaflet. Outer membrane has embedded proteins that play a variety of roles in virulence, including attachment and uptake into host cells. Lipids are composed of dimethylphosphatidylethanolamine, phosphatidylethanolamine, phosphatidylglycerol, cardiolipin, and phosphatidylcholine (Finnerty et al., 1979). These phospholipids in bacterial cell envelop is considered as permeability barrier but intriguingly, the removal of phosphatidylcholine from *L. pneumophila’s* envelope led to decreased cytotoxicity and reduced bacterial yield within macrophages (Conover et al., 2008). Moreover, absence of this lipid effected binding efficiency of bacteria to macrophages. Sawada et al. reported that *L. pneumophila* LPS specifically binds with pulmonary surfactant protein of lungs which play important role in innate immunity. This interaction leads to localization of *L. pneumophila* in lysosome and subsequently inhibition of bacterial intracellular growth (Sawada et al., 2010).

In conclusion, as outer membrane lipids participate in OMVs biogenesis, virulence mechanism and activation of host receptors by PRRs, studying *L. pneumophila* OMVs lipid is equally important than any other cargo to understand the contribution of OMVs in host-pathogen interaction.

#### 5.6.2. DNA cargo

In *L. pneumophila*, no research has been done yet on the OMV-associated DNA. Several investigations have reported the presence of DNA in OMVs fraction. Although the actual route through which DNA incorporates into OMVs is still not clear [37–39], literature shows that these OMV-associated DNA serves important role in horizontal DNA transfer and transfer of several functions in bacterial communities including antibiotic resistance, virulence, degradation (Supplementary Table S1) (Klieve et al., 2005; Rumbo et al., 2011; Velimirov and Hagemann, 2011; Fulsundar et al., 2014; Kulkarni et al., 2015; Chatterjee et al., 2017; Qiao et al., 2021). Studies on extracellular bacterial OMVs confirmed the presence of virulence genes in the DNA profile of OMVs also found transfer and expression of these genes in recipient bacteria (Yaron et al., 2000). However, such transfer between pathogenic bacteria and eukaryotic host is understudied. To date, there is only one study on *P. aeruginosa* which describes that OMVs have potential to deliver DNA to eukaryotic cells (Bitto et al., 2017). Regarding the direct influence of OMV-packaged DNA in host-pathogen there are few studies and needs to be further elucidated. As bacterial RNA and DNA are recognized by host endosomal nucleic acid receptors TLR7, 8, and 9 respectively (Jurk et al., 2002; Latz et al., 2004; Eigenbrod and Dalpke, 2015), so OMV associated DNA activate immune response by inducing Toll-like receptor 9 (TLR9) signaling (Perez Vidakovics et al., 2010; Bitto et al., 2021). A study in 2021 reported that *S. aureus* membrane vesicles were able to induce TLR9 suggesting that OMVs may contain immunostimulatory DNA.

## 6. *Legionella pneumophila* OMVs and host immune response

It has been demonstrated that OMVs of pathogenic bacteria promote the development of infection and host inflammation (Craven et al., 1980; Fiocca et al., 1999; Ren et al., 2012; Jung et al., 2016). OMVs can interact with a wide variety of immune cells and induce immunological responses (Kaparakis-Liaskos and Ferrero, 2015). Despite the fact that OMVs can cause inflammation in a variety of host tissues, the underlying processes are unclear. During infection, *L. pneumophila* interact with lung epithelial cells and lung macrophages. The epithelial surface containing resident immune cells, is the first line of defence. The interaction of OMVs with epithelial cells and macrophages not only induces cytokines but also stimulates PRR signaling. This is because OMVs contain numerous microorganism-associated molecular patterns (MAMPs), including RNA, DNA, LPS, peptidoglycan, and lipoproteins. MAMPS engage host PRRR and start the pro-inflammatory signaling chain. Since OMVs from different bacterial species change in their composition and content, so do the processes by which they trigger PRR signaling. For example, the LPS content of *E. coli* OMVs interacts with toll-like receptor 4 (TLR4) of human epithelial cells and drives TH4-dependent CXCL8 production (Soderblom et al., 2005), while *L. pneumophila* LPS poorly recognize TLR4 as lipid A of *L. pneumophila* contains unusual long, branched-chain fatty acids (Zähringer et al., 1995). Instead in *L. pneumophila* peptidoglycan-associated lipoproteins (PAL) are recognized by TLR2 (Liu and Shin, 2019).

Pneumonia induced by *L. pneumophila* is characterized by acute lung damage and severe hypoxemia. Patients exhibit elevated levels of inflammatory cytokines, including TNF, IFN-, IL-12, IL-6, IL-8, and granulocyte-colony stimulating factor, and disease severity is closely associated with the intensity of these inflammatory responses. To investigate the role of *L. pneumophila* OMVs in the inflammatory responses during bacterial infection, Galka et al. conducted a study where they examined the cytokine profiles of alveolar macrophages after a 15-h incubation with *L. pneumophila* OMVs (Galka et al., 2008). In comparison to the cytokine profiles observed during *L. pneumophila* infection, which included the induction of IL-2, IL-4, IL-6, IL-8, IL-17, IL-1β, INF-γ, MCP-1, TNF-α, and G-CSF, the OMVs were found to upregulate CCL2, CXCL8, G-CSF, IFNβ, IL6, IL7, and IL13. This highlights that IL-7 and IL-13 is a common secretion (Schmeck et al., 2007). The cytokine response induced by OMVs was found to be both dose-dependent and time-dependent in certain studies. In investigations involving *L. pneumophila* OMVs and a macrophage cell line called THP1, an increase in cytokine induction, including IL-8, IL-6, IL-10, TNF-α, and IL-1β, was observed as the concentration of OMVs increased. Additionally, this cytokine secretion was significantly higher at 48 h compared to 24 h. Using murine BMDM macrophages, similar time and dose dependent increase was observed for the CXCL1 cytokine (Jung et al., 2016).

Taken together*, L. pneumophila* OMVs have shown immunomodulatory potential when studied in epithelial cells A549, macrophage cell line THP-1, and murine BMDM suggesting the contribution of OMVs in patients’ inflammatory profile. However, the exact mechanism, and responsible cargos behind these immune modulations are largely unknown.

## 7. Potential applications of OMVs

### 7.1. OMV-based vaccine

OMVs are highly desirable as candidate vaccines due to their numerous inherent qualities. One of the key advantages is their exceptional stability even when exposed to different temperatures and treatments. Additionally, OMVs contain a variety of immunogenic membrane-associated and cytoplasmic components of their originating bacterium, are non-replicative, and thus safe (Arigita et al., 2004). Furthermore, the particulate nature of OMVs allows them to stimulate the innate immune system, resulting in their intrinsic adjuvant activity. This quality enables OMVs to enhance T-cell and antibody responses to antigens, making them even more effective (Etchart et al., 2006; Nieves et al., 2011; Reza Aghasadeghi et al., 2011). Finally, OMVs may be bioengineered to express any desired antigen and they can be modified to reduce their endotoxicity (Sanders and Feavers, 2011; Baker et al., 2014). This versatility makes OMVs particularly useful.

Despite of having good vaccination potential, OMV-based vaccines have been under development for more than two decades. However, significant progress has been made in the control of meningococcal serogroup B infection (Oster et al., 2005). This discovery of a vaccine with wide protective effectiveness against several *N. meningitidis* serogroup B isolates led to substantial advancement in the field. To date, 3 meningococcal vaccines are developed and commercialized. The VA-MENGOC-BC vaccine, developed and tested by the Finlay Institute in Cuba during an outbreak between 1987 and 1989, demonstrated 83% effectiveness after 16 months in young adults (Sierra-González, 2019). Similarly, the MenBvac vaccine, developed and evaluated by the Norwegian Institute of Public Health during an outbreak from 1988 to 1991, showed 87% effectiveness after ten months (Bjune et al., 1991). Another vaccine, MeNZB, was developed between 2004 and 2008 through collaboration between various institutions and was proven to be 73% effective in young adults. The most recent OMV-based vaccine, Bexsero, manufactured by Novartis and approved by the European Medicines Agency, comprises three recombinant antigens and detergent-extracted OMVs from the New Zealand strain (Semchenko et al., 2019). For use against gram-negative infections, more OMV-based vaccines are presently being created, although none of them have reached the clinical trial stage.

### 7.2. OMV based therapeutics – drug delivery

Early in the 1890s, cancer patients were treated with weakened bacteria as they stimulate anti-tumor cytokines CXCL10 and interferon-gamma. However, the safety concerns of using bacterial components have limited the clinical application of bacteria-mediated cancer therapy (Patyar et al., 2010; Hirayama and Nakao, 2020). Deletion of the msbB gene in *Salmonella* led to the reduction of immunotherapy’s side effects. The use of msbB-mutant *Salmonella* can protect the animal from septic shock caused by lipid A-stimulated tumor necrosis factor (TNFα) (Low et al., 1999). Although an impressive antitumor impact was seen in an animal investigation, *Salmonella*-mediated antitumor was unsuccessful in phase I clinical trial because it did not sufficiently reduce tumor growth (Mi et al., 2019). These studies provide solid ground for the use of modified OMVs in cancer immunotherapy. Recently, it was demonstrated that OMVs originating from attenuated *E. coli* strains may effectively prevent tumor growth and be employed as therapeutic agents to treat cancer. To reduce the negative effects of bacterial endotoxin lipopolysaccharide, the lipid A acyltransferase - (*msbB*) gene in *E. coli* was mutated. 12 h after the delivery of attenuated OMVs, a significant fluorescence signal was seen at the tumor location (Kim et al., 2017). As OMVs have an innate potential to protect their cargo, recent studies have reported effective drug delivery by OMVs. Successful drug loading into the OMV lumen has been achieved using electroporation. Allan and Beveridge in 2003 demonstrated that gentamicin loaded *P. aeruginosa* (PAO1 strain) OMVs hold the potential for treating Cepacia syndrome caused by *Burkholderia cepacia* (Allan and Beveridge, 2003).

## 8. Conclusion

The knowledge about function and fate of *L. pneumophila* OMVs inside host is still in the early stages, but available literature suggests its functional role in disease pathogenesis through OMVs attachment and uptake by host cells. However, there is a need for reliable and standardized methods to analyse the mechanisms by which OMVs enter and behave inside host cells. Furthermore, OMVs carry a variety of virulence factors which provides an intriguing hypothesis regarding the delivery of these factors to host cells. To gain more insights into this process, further research is needed, particularly through intracellular infection studies. Additionally, the variety of cargo carried by OMVs highlights the importance of studying individual components to understand their contributions to disease development. While some studies have been conducted on protein and RNA components in *L. pneumophila*, limited research has focused on other components, which leaves their role in pathogenesis uncertain. Lastly, understanding the potential benefits of *L. pneumophila* OMVs is crucial, as they may have ability to play a dual role in the intracellular infection cycle. On one hand, they can help bacteria by delivering virulence factors, thereby promoting their virulence. However, on the other hand, these OMVs also carry immunomodulatory properties that could be beneficial to the host by potentially triggering a protective immune response. In first case scenario, by targeting specific components within the OMVs that are involved in their interaction with human lung cells or macrophages, novel treatment approaches for this infection may be developed. For the second case scenario, immunomodulatory properties of OMVs could be utilized for the development of vaccines for protecting patients from bacterial infections.

In summary, the discovery of OMVs has significantly advanced our understanding of bacterial physiology. Despite the existing challenges, we anticipate that future research including the characterization of *L. pneumophila* OMVs produced during infection and their interactions with the host, along with investigations of the mechanisms of vesicle secretion, will deepen our knowledge about bacterial strategies and host defence responses. Finally, exploring *L. pneumophila* OMVs may guide us in the development of innovative immunization approaches and predictive biomarkers for treatment and vaccine efficiency.

## Author contributions

AA: Conceptualization, Writing – original draft. FC: Conceptualization, Visualization, Writing – review & editing. PL: Funding acquisition, Supervision, Validation, Visualization, Writing – review & editing.
