# Supplementary material for: Role of Legionella pneumophila outer membrane vesicles in host-pathogen interaction
Source: Front Microbiol. 2023 Sep 25;14:1270123. doi: 10.3389/fmicb.2023.1270123 (PMC10561282; doi:10.3389/fmicb.2023.1270123)
Supplement: Supplementary file 1 [file Table_1.pdf]

(Supplementary Table 1). Published studies on OMV-mediated gene transfer

| **OMVs Donor/s** | **Recipient/s** | **Nature of DNA being transferred by OMVs** | **Function transferred** | **Reference** |
|---|---|---|---|---|
| | | **Inter and intra-species** | | |
| AR67- *Wild type Ruminococcus albus* strain And YE73- *Ruminococcus* sp. strain | Cel⁻ mutants of strain of *B. fibrisolvens* and AR5YE71-2 and YE71-11 | linear dsDNA | Cellulolytic capabilities (ability to degrade crystalline cellulose) | (1) |
| Environmental vesicle DNA | Amino acid deficient strain *E. coli* AB1157 | 370 kbp DNA | Restored growth of *E. coli* auxotroph | (2) |
| Transformed DH5α *E. coli* (with Pet28a plasmid) | *E. coli* BL21 | Pet28a plasmid containing *nirS* gene | nitrite reductase activity. | (3) |
| Transfected *E. coli* O157:H7 (with *pGFP plasmid)* | *S. enterica and* *E. coli* JM109 | 1. Gfp plasmid with ampicillin resistance gene<br>2. Phage DNA Stx | Antibiotic resistance to ampicillin, green fluorescent colonies<br>Increased stx mediated cytotoxicity | (4) |
| *E. coli* | *A. radioresistens* and *P. aeruginosa* and | melittin and colistin resistance genes | Colistin and melittin resistance | (5) |
| *A. baumannii* clinical strains AbH12O-A2 and AbH12O-CU3) | *A. baumannii* ATCC 17978 (carbapenem-susceptible) | plasmid-borne OXA-24 carbapenemase Gene | Full resistance to carbapenems | (6) |
| *A. baumannii* (with New Delhi metallo-β-lactamase-1 (NDM-1) gene plasmid) | *A. baumannii*<br>*E. coli* | $bla_{NDM-1}$-bearing plasmid | Increased MIC values for β-lactam antibiotics | (7) |
| *Acinetobacteri baylyi* JV26 strain with pMU125 plasmid (containing GFP and beta-lactamase against ampicillin) | Mutant *A. baylyi* JV26, *E. coli* DH5α | Co-transfer of the *bla* and *gfp* genes with intact plasmid. | Resistance to ampicillin<br>Green fluorescent colonies | (8) |
| *P. gingivalis* mutant (FimA gene Incorporated with erythromycin-resistant gene.) | *P. gingivalis* | FimA and Sod gene (superoxide dismutase) | resistance to erythromycin | (9) |
| | | **Bacteria to Eukaryotic host** | | |
| *P. aeruginosa* PA103 | A549 lung epithelial cells | 859 bp *P. aeruginosa* *narG* gene - (detected within A549 nuclear fraction by PCR) | Not Tested | (10) |

## References


1. Klieve AV, Yokoyama MT, Forster RJ, Ouwerkerk D, Bain PA, Mawhinney EL. Naturally occurring DNA transfer system associated with membrane vesicles in cellulolytic Ruminococcus spp. of ruminal origin. Applied and environmental microbiology. 2005;71(8):4248-53.
2. Velimirov B, Hagemann S. Mobilizable bacterial DNA packaged into membrane vesicles induces serial transduction. Mobile genetic elements. 2011;1(1):80-1.
3. Qiao W, Wang L, Luo Y, Miao J. Outer membrane vesicles mediated horizontal transfer of an aerobic denitrification gene between Escherichia coli. Biodegradation. 2021;32(4):435-48.
4. Yaron S, Kolling GL, Simon L, Matthews KR. Vesicle-mediated transfer of virulence genes from Escherichia coli O157: H7 to other enteric bacteria. Applied and environmental microbiology. 2000;66(10):4414-20.
5. Kulkarni HM, Nagaraj R, Jagannadham MV. Protective role of E. coli outer membrane vesicles against antibiotics. Microbiological research. 2015;181:1-7.
6. Rumbo C, Fernández-Moreira E, Merino M, Poza M, Mendez JA, Soares NC, et al. Horizontal transfer of the OXA-24 carbapenemase gene via outer membrane vesicles: a new mechanism of dissemination of carbapenem resistance genes in Acinetobacter baumannii. Antimicrobial agents and chemotherapy. 2011;55(7):3084-90.
7. Chatterjee S, Mondal A, Mitra S, Basu S. Acinetobacter baumannii transfers the blaNDM-1 gene via outer membrane vesicles. Journal of Antimicrobial Chemotherapy. 2017;72(8):2201-7.
8. Fulsundar S, Harms K, Flaten GE, Johnsen PJ, Chopade BA, Nielsen KM. Gene transfer potential of outer membrane vesicles of Acinetobacter baylyi and effects of stress on vesiculation. Applied and environmental microbiology. 2014;80(11):3469-83.
9. Ho M-H, Chen C-H, Goodwin JS, Wang B-Y, Xie H. Functional advantages of Porphyromonas gingivalis vesicles. PloS one. 2015;10(4):e0123448.
10. Bitto NJ, Chapman R, Pidot S, Costin A, Lo C, Choi J, et al. Bacterial membrane vesicles transport their DNA cargo into host cells. Scientific reports. 2017;7(1):1-11.
